# Calibration Update and Drift Correction for Electronic Noses and Tongues

**DOI:** 10.3389/fchem.2018.00433

**Published:** 2018-09-25

**Authors:** Alisa Rudnitskaya

**Affiliations:** Centre for Environmental and Marine Studies CESAM and Chemistry Department, University of Aveiro, Aveiro, Portugal

**Keywords:** calibration update, calibration transfer, drift correction, electronic nose, electronic tongue

## Abstract

One of the obstacles to the wider practical use of the multisensor systems for gas and liquid analysis—electronic noses and tongues, is the limited temporal validity of the multivariate calibration models. Frequent recalibration of multisensor systems is often excessively costly and time consuming due to the large number of necessary reference sample and their limited availability. There are several circumstances that can invalidate multivariate calibration model. The most common problem in the case of sensor systems is temporarily drift or gradual change of sensor characteristics occurring during sensor exploitation. Another common situation is a change in the composition of the analyzed samples that also alters sensor response due to the matrix effects. Finally, a necessity to replace sensors in the array or to transfer calibration model from one sensor set or one type of sensors to the other can arise. As an alternative to the recalibration of the sensor system using full set of calibration samples, drift correction and calibration update has been proposed. The main approaches can be summarized as follows:

Drift correction that consists in modeling sensor temporarily drift or drift direction using a series of measurements and then using it for correcting new data.
Calibration standardization that aims to correct new measured data by eliminating new variation. For this purpose, a relationship between two experimental conditions is established using a reduced set of samples measured at both conditions (standardization subset).
Calibration update that consists in incorporation of new sources of variance in the calibration model by recalculating it using initial calibration samples and reduced set of samples measured at new conditions. The latter can be either standard or unknown samples.

This paper presents an overview of different methods reported for the drift correction and calibration update of the electronic noses and tongue and discussion of the practical aspects of their implementation.

## Introduction

The electronic noses and electronic tongues are multisensor systems based on arrays of cross-sensitive or partially selective chemical sensors and data processing tools. They have been shown to be promising analytical instruments for a wide range of applications including environmental, food and clinical analysis among others (Schaller et al., 1998; Winquist et al., 2002; Legin et al., 2003).

Chemical sensors as any analytical instruments require regular calibration to account for the changes in their response and ensure their proper functioning. Alterations in the instrumental response may result from the changes of the environmental conditions, composition of the measured samples or device characteristics. The latter is of particular relevance for the chemical sensors that are prone to gradual change of their characteristics or temporarily drift occurring during their exploitation. While regular re-calibration using standards is an established procedure for the individual sensors, as e.g., calibration of pH glass electrode using a series of buffer solutions, it becomes problematic in the case of multisensor systems. Both electronic noses and tongues include partially specific sensors that produce non-selective signals in the multicomponent media, such as almost all real world samples. Therefore, they rely on multivariate calibration models for interpreting their responses and relating them to concentration or property of interest. Multivariate calibration requires large number of standard samples, some of which could be of limited availability. Consequently, frequent recalibration of sensor arrays is prohibitively costly both with respect to the necessary time, availability of standard samples and labor. Alternatively, calibration transfer or update requiring small number of standard samples or no standard samples at all can be implemented.

Impracticability of re-calibration also applies to other analytical techniques that produce non-specific signals in multicomponent samples, the most common example of these being near infrared (NIR) spectroscopy. As NIR spectroscopy is widely applied to the industrial monitoring, significant efforts have been directed to the development of the calibration transfer and update techniques for the NIR spectroscopic instruments (Feudale et al., 2002). However, fewer works addressed this issue for the chemical sensor arrays. In the field of gas sensor arrays main efforts have been directed to the drift filtering and compensation (Marco and Gutierrez-Galvez, 2012; Deshmukh et al., 2015; Liang et al., 2018) while very few papers dealt with calibration update for the electronic tongue systems.

It should be noted that several measures have been recommended to prolong validity of the calibration models. These include optimization of the sensor manufacturing procedures and tighter sensor quality control, which improve stability of the sensing layer, and development of the measuring procedures, including sensor cleaning and conditioning for sensing surface regeneration, and controlled measuring conditions, which improve repeatability of the sensor signal (Korotcenkov and Cho, 2011). Notwithstanding importance of these factors for the proper functioning of the sensors, in practice they are not sufficient to completely avoid recalibration.

This paper will focus exclusively on the calculation based techniques of the drift reduction and calibration update and will present an overview of different methods reported for the drift compensation, calibration update and transfer for the electronic noses and tongues and discussion of the practical aspects of these methods' implementation. Described methods are summarized in the Table 1.

**Table 1:** Summary of calibration transfer and drift reduction methods.

**Method**	**Sensor technology**	**Task**	**Model**	**N transfer samples**	**References**
**COMPONENT CORRECTION**					
PCA	MOX gas sensors Voltammetric metal liquid sensors	Classification, recognition Recognition			Artursson et al., 2000; Holmin et al., 2001
ICA	QMB, MOX and polymeric gas sensors	Recognition	PCA		Di Natale et al., 2002; Kermit and Tomic, 2003
CPCA	Polymeric gas sensors	Classification, recognition			Ziyatdinov et al., 2010
CCA and PLS	MOX gas sensors	Classification			Gutierrez-Osuna, 2000
OSC	Polymeric gas sensors	Classification	k-NN		Padilla et al., 2010
**DRIFT MODELING**					
DWT	MOX gas sensors	Recognition	PCA		Zuppa et al., 2007
ARMA	MOX gas sensors	Sensor response			Zhang and Peng, 2016
Kalman filter	MOX gas sensors	Drift prediction			Zhang and Peng, 2016
Chaotic time series	MOX gas sensors	Drift prediction			Zhang et al., 2013
**SIGNAL STANDARDIZATION–CALIBRATION TRANSFER BETWEEN INSTRUMENTS**					
SWS	Potentiometric liquid sensors	Quantification	PLS	10	Khaydukova et al., 2017b
DS + PLS	QMB gas sensors	Recognition	PCA	72	Tomic et al., 2002
DS + Robust regression	MOX gas sensors	Classification	ANN	27	Deshmukh et al., 2014
DS + MLR	Polymer gas sensors MOX gas sensors Potentiometric and voltammetric liquid sensors Potentiometric liquid sensors	Classification Quantification Quantification Quantification	DFA SVR PLS PLS	8 5 3 10	Balaban et al., 2000; Fonollosa et al., 2016; Khaydukova et al., 2017a,b
DS + ANN	QMB and polymer gas sensors	Recognition	PCA	138	Shaham et al., 2005
PDS + MLR	MOX gas sensors	Quantification	SVR PLS	5 5	Fernandez et al., 2016; Fonollosa et al., 2016
WPDS + SEMI + robust regression	MOX gas sensors	Classification and quantification		6	Yan and Zhang, 2015
DS + RWLS	MOX gas sensors	Quantification	BPNN	5	Zhang et al., 2011
Tikhonov regularization	Potentiometric liquid sensors	Quantification	PLS	10	Khaydukova et al., 2017b
**SIGNAL STANDARDIZATION–CALIBRATION UPDATE**					
SWS	Potentiometric liquid sensors	Classification and quantification	LDA, LR, PLS-DA, PLS	3 10	Sales et al., 1999; Panchuk et al., 2016
DS + MLR	Potentiometric liquid sensors	Classification and quantification	LDA, LR, PLS-DA, PLS	3	Panchuk et al., 2016
DS + PLS	Potentiometric liquid sensors	Quantification	PLS	4–7	Rudnitskaya et al., 2017
DS + ANN	Potentiometric liquid sensors	Quantification	PLS	4–7	Rudnitskaya et al., 2017
PDS + PLS	Potentiometric liquid sensors	Quantification	PLS	3–10	Sales et al., 1999, 2000
**MODEL EXPANSION–CALIBRATION TRANSFER**					
TCTL TCTL + SEMI	MOX gas sensors	Classification and quantification	LR, RR	6	Yan and Zhang, 2016a
**MODEL EXPANSION–CALIBRATION UPDATE**					
Weighting	Potentiometric liquid sensors	Quantification	PLS	4–7	Rudnitskaya et al., 2017
Tikhonov regularization	Potentiometric liquid sensors	Quantification	RR	4–7	Rudnitskaya et al., 2017
Joint-Y PLS	Potentiometric liquid sensors	Quantification	PLS	4–7	Rudnitskaya et al., 2017; Cruz et al., 2018
TCTL TCTL + SEMI	MOX gas sensors	Classification and quantification	LR, RR	10	Yan and Zhang, 2016a
DAELM-S	MOX gas sensors	Classification		20–30	Zhang and Zhang, 2015
SAELM-T	MOX gas sensors	Classification		40–50	Zhang and Zhang, 2015
DCAE	MOX gas sensors	Classification		10	Yan and Zhang, 2016b
**ADAPTIVE MODEL EXPANSION**					
SOM	MOX gas sensors Simulated data with drift	Classification Classification	SOM SOM		Di Natale et al., 1995; Marco et al., 1998
mSOM	Polymeric gas sensors	Classification	mSOM + LVQ		Distante et al., 2002; Zuppa et al., 2004
A^2^INET	MOX gas sensors and simulate data	Classification	k-NN		de Castro and von Zuben, 2002; Martinelli et al., 2013, 2014
Unsupervised selection	QMB gas sensors	Classification	LDA		Magna et al., 2018
Semi-boost, COREG	MOX gas sensors	Classification	BPNN		De Vito et al., 2012
System identification	MOX gas sensors	Classification	Box-Jenkins model + recursive LS	150	Holmberg et al., 1996, 1997
Classifier ensembles	MOX gas sensors	Classification	SVM		Vergara et al., 2012
**GLOBAL CALIBRATION**					
Fuzzy inference system	MOX gas sensors	Quantification	PLS		Šundić et al., 2002
	MOX gas sensors	Classification	PLS-DA + k-NN		Solórzano et al., 2018

## Factors invalidating calibration models

There are several circumstances that can invalidate multivariate calibration model comprising changes in the sensor characteristics due to the drift or sensor replacement, changes of the environmental conditions or composition of measured samples. The most common problem in the case of sensor systems is temporarily drift or gradual change of sensor characteristics occurring during its exploitation. Causes of the drift vary depending on the employed transducer and sensing material with each type of sensor having its particular Achilles' heel.

Conductometric metal oxide gas sensors (MOX) are the most commonly applied in the electronic nose systems due to their low cost and sensitivity to a wide range of gases (Meixner and Lampe, 1996). Sensing layer of MOX sensors is n-type semiconducting metal oxides, of which tin dioxide is the most common. Sensing mechanism of MOX sensors is based on catalytic oxidation of analyte gases on the sensing layer consisting of contiguous small metal oxide grains. Oxygen absorption by the grains creates depletion layer on their surface, increasing their resistance and, consequently, resistance of the entire sensing layer. MOX sensors respond to the volatiles capable of absorbing and undergoing red-ox reaction on the sensor surface. Stability of the sensor response is thus conditioned by the two main effects: changes of the morphology of the sensing layer and its poisoning (Korotcenkov and Cho, 2011). Structural changes may include changes of the size and geometry of the metal oxide grains leading to the alterations in their conductivity and catalytic properties. Cracking of the metal oxide film after large number of operation cycles (Sharma et al., 2001) and phase separation between metal oxide and additives when they are used are other factors affecting sensor stability (Wang et al., 2007). Finally exposure to the compounds capable to irreversibly bind to metal oxides results in the inhibition of the catalytic activity or poisoning (Meixner and Lampe, 1996; Pijolat et al., 1999). Nitrogen, phosphorus and sulfur containing compounds are typical examples of such inhibitors.

Another group of sensing materials commonly employed in the electronic noses are conducting polymers, such as polypyrrole (PPy), polyaniline (PANI), polythiophene (PTh) and their derivatives (Bai and Shi, 2007; Bernabei et al., 2016). Wide adoption of intrinsically conducting polymers in gas sensing is due to their high sensitivity, good mechanical properties allowing for easy sensor device manufacturing and low operation temperature. Conducting polymers also prone to temporal changes of both baseline and sensitivity (Schaller et al., 2000; Kondratowicz et al., 2001; Kemp et al., 2006). Irreversible changes of response are typically attributed to de-doping and consequent decrease of conductivity of the polymer, which can be provoked by the nucleophilic attack on the carbon back backbone by some volatile compounds (Kondratowicz et al., 2001; Kemp et al., 2006) or oxidation (Schaller et al., 2000). Similar process i.e., partial conversion of the polymer from electrically conducting into non-conducting state have been identified as a source of irreproducibility of potentiometric chemical sensors employing conducting polymers as a solid inner contact (Lindfors and Ivaska, 2004; De Marco et al., 2008, 2009; He et al., 2015). Polymer conversion in this case is caused by the protonation/deprotonation and red-ox reactions occurring in the water layer forming between sensitive membrane and inner solid contact (De Marco et al., 2008).

Two main types of sensors employed in the electronic tongues are potentiometric and voltammetric. Voltammetry is based on the measurements of current generated by reduction and oxidation of species on the electrode surface. Fouling of the electrode surface by the reaction products leads to its inhibition and frequent renewal of the electrode surface is requires to maintain its active state (Štulík, 1992). Mechanical, thermal and electrochemical pre-treatment procedures were shown to be effective for the restauration of the electrode surface properties (Holmin et al., 2004; Olsson et al., 2006). Thorough polishing of the metal electrodes is effective for restoration of electrode surface and drift removal (Cavanillas et al., 2015). When mechanical polishing is not feasible, i.e., in the case of thin film sensors, flow-through set-ups, etc., mathematical methods to account for the drift are necessary (Holmin et al., 2001).

Fouling is less critical for the potentiometric chemical sensors that function in the zero-current measuring set-up, however, it may still occur upon sensor exposure to certain substances. For example, poisoning of the solid sensors through formation of insoluble compounds on their surface or absorption of lipophilic compounds by the polymeric membranes have been reported (Vlasov and Bychkov, 1987; Lisak et al., 2016). Potentiometric sensors with polymeric membranes, mainly plasticized polyvinyl chloride (PVC), typically display rapid drift of their signal during first days of their use, which is attributed to the equilibration processes between water and membrane phases. Further, gradual leaching of the active compounds, ionophores, from the sensing membrane, may induce low long-term drift (Lindfors and Ivaska, 2004). In the potentiometric sensors with glass membranes potential-generating processes occur at the interface between solution and modified surface layer, which is formed as a result of oxidation and partial destruction of glass network by solution (Vlasov and Bychkov, 1987). Interaction with compounds present in the analyzed media may lead to changes in the modified surface layer, which provokes alteration of the sensor response or drift (De Marco et al., 2003).

Another factor affecting sensor characteristics is fluctuations of the temperature and humidity in the environment, in which sensors are deployed. Effect of these two parameters is especially pronounced in the case of gas sensors. In particular, at some temperature ranges change of the baseline conductivity of conducting polymer sensors provoked by the change of the temperature for 1°C may be comparable of the sensor response to the analyte (Schaller et al., 2000). Liquid sensors are less sensitive to the temperature fluctuations. For example, while response of potentiometric sensors is dependent on the temperature according to the Nernst equation, potential changes provoked by temperature alterations within ca. 4°C are considered negligible, therefore less strict temperature control during measurements is required.

Clearly as drift causes are different, its manifestation in the sensor system response will also differ. Therefore, several strategies were put forward to deal with sensor drift.

## Drift correction for known interferences

When factors causing sensor drift are not only known but can also be easily quantified, as is the case with effects of humidity and to the less extent temperature fluctuation on the MOX gas sensors response, these factors can be measured and used to compensate for drift they induce. Temperature and humidity sensitivity of the sensors may be calculated and used for sensor signal correction (Kashwan and Bhuyan, 2005; Hossein-Babaei and Ghafarinia, 2010; Mumyakmaz et al., 2010; Huerta et al., 2016).

## Drift compensation and modeling

Drift compensation and modeling methods presume that drift can be separated from the analytical signal and modeled and obtained model can be used for the correction of the sensor array response in new samples.

Group of methods called Component Correction (CC) is based on the assumption that sensors of the array have similar (correlated) behavior with the respect to drift and drift of sensor array has a specific direction, which is the same for all measured samples and reference gas. Therefore, drift correction can be done by identifying and modeling drift direction in the reference samples and subtracting it from the new data. CC was implemented using a number of techniques with PCA and Partial Least Square regression (PLS) being the most common (Artursson et al., 2000; Gutierrez-Osuna, 2000).

A very simple linear drift correction of sensor signals using regularly measured standard sample has been proposed in Haugen et al. (2000). This approach was tested on the data of the monitoring of fish and milk spoilage using the MOX sensor array. The advantage of this method is its obvious simplicity, though for its proper functioning sensor drift should be linear over time.

PCA applications to drift modeling is straightforward: if the sensor responses in the reference samples have significant drift, the first components in a PCA model calculated using only measurements in reference sample will describe the direction of the drift (Artursson et al., 2000). Therefore, loading vector p_d_ of the PCA model calculated in reference sample is attributed to the noise and used to calculate projection t_d_ of the new measurements X_n_. Drift correction is performed by subtracting drift component from the new measurement matrix:

(3.1) $${\text{X}}_{\text{n}}^{\text{corr}}={\text{X}}_{\text{n}}-{\text{t}}_{\text{d}}{\text{p}}_{\text{d}}^{\prime }$$

Similar reasoning is underlying PLS application for drift compensation but instead of considering direction of the maximum variance of the sensor responses in the reference samples as a drift, changes in the sensor array response in reference samples are modeled as a function of time. PLS model is calculated using sensor responses in the reference samples as an X_d_ matrix and time as Y matrix. Resulting loadings p_d_ and weights w_d_ are used to calculate projection of new measurements:

(3.2) $${\text{t}}_{\text{d}}={\text{X}}_{\text{n}}{\text{w}}_{\text{d}}{\left({\text{p}}_{\text{d}}^{\prime }{\text{w}}_{\text{d}}\right)}^{-1}$$

New measurements are corrected for drift by extracting drift component:

(3.3) $${\text{X}}_{\text{n}}^{\text{corr}}={\text{X}}_{\text{n}}-{\text{t}}_{\text{d}}{\text{p}}_{\text{d}}^{\prime }$$

Both PCA and PLS model for drift correction may include one or several latent variables. Important issues for the CC are scaling and transformation, which must be the same for both reference and analyzed samples. Outliers, which can skew drift direction, should be detected and removed prior to the drift model calculations.

CC has been successfully applied to the drift correction of MOX sensor array exposed to the 4 gases (hydrogen, ammonia, ethanol, and ethene) and their mixtures during 2 months period. Mixture of 4 gases at their mean concentration levels was measured throughout experiment as a reference. Both recognition and classification performance of the electronic nose was improved after drift correction compared to the uncorrected or corrected by multiplicative drift correction data (Artursson et al., 2000).

CC is based on an assumption that drift directions of sensors in reference gas and all measured samples are highly correlated. If this assumption does not hold, drift correction will be inefficient and, furthermore, some analytical information will be removed together with drift. A generalization of PCA to several classes called Common Principal Component Analysis (CPCA) has been proposed to take into account different behavior of sensors in different samples (Ziyatdinov et al., 2010). CPCA calculates loading vector p so that it expresses common covariance for all classes (gases) instead of variance observed in the reference gas. Detailed description of CPCA can be found in (Flury, 1984). CPCA was applied to the drift correction of the data set measured in ammonia, n-butanol and propanoic acid at different concentration levels by an array of 17 polymeric sensors over the period of 7 months. The first 1,000 and 1,200 measurements out of 3,484 were used for drift modeling by CPCA and PCA. Better results were obtained using larger calibration data set with CPCA performing better than both PCA and uncorrected data.

Drift correction using PLS and Canonical Correlation Analysis (CCA) that employs both measurements in washing and reference gas sample has been described in Gutierrez-Osuna (2000). Drift reduction algorithm consisted in three steps:

1. Find linear projections $\tilde{\text{x}}$ and $\tilde{\text{y}}$ of measurements in wash and reference gas, x, and samples, y, that are maximally correlated: {A, B} = argmax[ρ(A_x_B_x_)];
2. Fit a regression model ${\text{y}}_{\text{pred}}=\text{W}\tilde{\text{y}}$ by ordinary least squares:
  $$\text{W}=\arg\min{\left(\text{y}-\text{W}\tilde{y}\right)}^{2};$$
3. Deflate y and use the residual z as a drift corrected data for classification purposes:
  $$\text{z}=\text{y}-{\text{y}}_{\text{pred}}=\text{y}-\text{WBy}.$$

PLS and CCA were used to find projection matrices A and B in the first step of the algorithm. Drift correction was applied to the measurements made by an array of 10 MOX sensors in four spices' headspace during 3 months. Success of drift correction depended on the size of the calibration data set and on the period of time elapsed between last calibration measurement and test. Both PLS and CCA could maintain correct classification rate of 95% for up to 10 consecutive measurement sessions when at least 5 days of measurements were used for calibration. This was significant improvement in comparison to uncorrected data, for which classification rate varied between 70 and 80% in the same settings. However, increase of the time elapsed between calibration and test measurements was shown to have detrimental effect on the efficiency of drift correction.

CC using PCA was employed for the drift reduction of the voltammetric electronic tongue and compared to the additive correction (Holmin et al., 2001). Additive correction consisted in subtraction of the sensor signal measured in the reference sample from the signals measured in the analyzed samples. An electronic tongue comprising 4 metal electrodes (gold, iridium, platinum, and rhodium) was used for measurements in the green and black tea brews, apple juice and process water from pulp and paper plant. Solution of potassium hexacyanoferrate(II) in phosphate buffer was used as a reference solution for drift modeling. Both CC and additive drift correction were effective in drift reduction for the studied data set as sensor drift in this experiment was linear.

Modification of PCA called correlated information removing based interference suppression (CIRIS) has been suggested for background correction of the electronic nose in (Liang et al., 2017). CIRIS consists in applying PCA to the measurements in reference gas and analyzed samples individually. The 1st PC calculated in the reference gas describes to the interference/drift of the sensor signals and corresponding loading vector corresponds to the main direction of this interference. Loading vector calculated in the analyzed samples, which is most correlated with that interference direction, is used for data correction. CIRIS was applied for correction of measurements with an array of 30 tin oxide sensors in the headspace 8 cultured bacteria, which are commonly associated with wound infections. Headspace of aqueous ethanol solution was used as a reference gas. CIRIS improved correct classification rate compared to the uncorrected data from 85 to 93%.

Drift filtering using OSC has been reported in (Padilla et al., 2010). The main idea of OSC consists in removal of the variance not correlated to a vector (or matrix) Y. This is done by constraining the deflation of non-relevant information of X in such a way that only information orthogonal to Y is removed (Wold et al., 1998). OSC filtering was applied to the data set consisting of measurements with an array of 17 conductive polymer sensors in ammonia, propanoic acid and n-butanol over the period of 10 months. Measurements made during the first 15 days were used for calculation of OSC model and optimization of a number of components to remove, and for calculation of classification model using k-NN. Use of OSC filter permitted to maintain correct classification rate between 80 and 98% for the test data set compared to 64–93% for uncorrected data. PCA correction performed slightly worse than OSC with correct classification rate between 78 and 97% for test data.

Another method for compensation of the drift that has a specific direction is Independent Component Analysis (ICA) (Di Natale et al., 2002; Kermit and Tomic, 2003; Tian et al., 2012). Similarly to PCA, ICA decomposes matrix of sensor signals X as *X* = *AS*, where A is called mixing matrix and S is a matrix of independent components or source signals (Hyvärinen and Oja, 2000). ICA differs from the orthogonal methods such as PCA in that extracted latent variables are statistically independent, i.e., information contained in one independent component cannot be inferred from the others. In practice it means that matrix of sensor array signals can be decomposed into a series of components, some of which are correlated with analytical signal and some with drift. Therefore, independent components mostly correlated with known drift source can be removed.

Removal of independent components correlated with temperature and humidity fluctuations was demonstrated to improve capability of an arrays of quartz microbalance (QMB) sensors with metalloporphyrine membranes to discriminate between two types of peaches (Di Natale et al., 2002). Limitation of this work is that measurements were done during only 4 days.

The same approach was applied to the background removal from the responses of an array of 30 metal oxide gas sensors in the headspace of the infected mouse wounds (Tian et al., 2012). Signal correction by ICA was found to be more effective than PCA and permitted to improve correct classification rates for three bacterial species compared to uncorrected data from 85 to 96%.

ICA can be also used for removal of drift of unknown origin in unsupervised mode (Kermit and Tomic, 2003). After pre-processing of the raw electronic nose data by PCA, obtained square matrix with number of principal components equal to the number of sensor signals was decomposed by ICA. Some of the Independent Components are expected to be highly correlated with sensor drift while other components are expected to be largely free of drift and, thus, can be used for classification purposes. This approach was tested on the data from the mixed sensor array comprising 10 MOSFET and 12 MOS sensors. Sensor signals were collected in two measuring sessions: in headspace of solutions of 5 organic compounds (1% cyclohexanal, 1% ethanol, 5% ethanol, 1% hexanal, and 1% isopropanol) and 5 sample of propanol and butanol at different concentrations, with 200 and 90 measurements acquired, respectively. Unfortunately, no information about timeframe of the measurements was provided. Only sensors responding to some of the analytes were retained for the analysis, resulting in 9 and 6 variables for the 1st and 2nd measuring session, respectively. In both cases combination of PCA and ICA permitted to separate drift from analytical signal improving discrimination performance of the sensor array.

Another group of drift filtering methods is based on the premises that drift of a sensor array resides in low frequency of the signal contrary to the response to analytes, which is high frequency. Sensor signals are split into low and high frequency components, and the slowest signal component is considered drift and removed from the data.

Application of one of such methods - Discrete Wavelet Transform (DWT) was described in Zuppa et al. (2007). The procedure of the drift removal using DWT consist of three steps: decomposition of the signal, thresholding, and reconstruction of the signal. In the decomposition step, a signal is decomposed into a set of orthonormal wavelet functions that constitute a wavelet basis. After that small wavelet coefficient associated to the noise are remove and signal is reconstructed using remaining coefficients. Detailed description of DWT algorithm can be found in Mallat (1989); Ergen (2012). Efficiency of DWT for drift removal was demonstrated on the artificially generated drifting sensor responses.

Methods developed for time series analysis such as Autoregressive moving average (ARMA), Kalman filter (Zhang and Peng, 2016) and chaotic time series analysis (Zhang et al., 2013) have been adopted for drift modeling. These methods are used to analyze time series of signals, in which the present signal value depends on its preceding values. ARMA and Kalman filter algorithms have been tested on data set consisting of the same sample measured by an array of 4 MOX sensors for 10 months. Composition of measured samples was unfortunately unspecified. Measurements carried out during the first month of experiment were used to model drift of each sensor of the array sensor and the rest of the data for model testing. ARMA was found to be more efficient with prediction errors of sensor drift about an order of magnitude lower compared to Kalman filter. Detailed description of ARMA and Kalman filter algorithms can be found in Navarro-Moreno (2008) and Faragher (2012), respectively.

The same group has applied a chaotic time series analysis to the sensor drift modeling (Zhang et al., 2013). Drift extraction was done using discrete Fourier Transform with an assumption that sensor drift belongs to low frequency part of the signal. Further drift modeling included two steps: phase space reconstruction of the drift and training of the Radial Based Function (RBF) neural network for the prediction of the sensor baseline. This approach was tested on the data set described above. Increase of the number of measurements used for the modeling was demonstrated to improve baseline prediction. Using at least 1,000 points measured during 3 weeks period was enough to achieve the best performance in prediction of the sensor baseline for the data measured in the following ca. 9 months.

It is important to note that in Zhang et al. (2013) and Zhang and Peng (2016), drift modeling methods were applied to the prediction of the portion of the sensor signals separated as drift, efficacy of the methods of time series analysis to maintain initial calibration performance in prediction of new samples has not been demonstrated.

Obvious problem with drift modeling methods is that a relatively long series of measurements is required to produce a drift correction model. Measurements made during several weeks are typically used. Furthermore, it can be expected that after some time of the sensor system functioning, drift correction model would become invalid and would need an update, which logically requires another worth of several weeks of measurements.

## Multivariate calibration update

Calibration update methods relay on the regular sensor array measurements in a small number of standards that are used for correction of sensor responses in new unknown samples or for re-calculation or update of calibration model.

### Data standardization

This group of methods aims to correct new measured data by eliminating new variation. For this purpose, a relationship between two experimental conditions is established and measurements made in new experimental conditions are corrected by this relationship and used for concentration prediction in new samples using initial calibration model. Reduced set of standards measured at both conditions also called standardization or transfer data set is used to for such correction. Two approaches are possible for data standardization: correction of the concentrations predicted at new conditions correction of signals measured in the new conditions.

#### Slope and bias correction of the predicted values

The slope and bias correction adjusts concentrations predicted in unknown samples using a relationship between concentrations predicted by the calibration model in the standardization subset measured initially and in new conditions (Sales et al., 1999). The relationship between two sets of prediction concentrations, c_i_ and c_n_, corresponding to the initial and new conditions, respectively, is calculated by the univariate regression:

(4.1) $${\text{c}}_{\text{i}}\text{=Intercept}+\text{Slope}\times {\text{c}}_{\text{n}}$$

Concentrations of the samples measured in new conditions are predicted using initial calibration model and corrected using slope and bias determined using update sample set as described above:

(4.2) $${\text{c}}_{\text{n}}^{\text{corr}}\text{=Intercept}+\text{Slope}\times {\text{c}}_{\text{n}},$$

where ${\text{c}}_{\text{n}}^{\text{corr}}$ is corrected concentrations measured in new conditions.

Slope and bias correction has been applied to the data from the potentiometric sensor arrays: an array of 7 sensors with plasticized PVC membranes used for quantification of potassium and calcium in the synthetic ground water (Sales et al., 1999) and an array of 7 sensors with chalcogenide glass and plasticized PVC membranes used for quantification of copper and lead in the model solutions (Rudnitskaya et al., 2017). In both cases slope and bias correction was compared to the other calibration update techniques and was found to be the least less efficient (Sales et al., 1999; Rudnitskaya et al., 2017).

#### Signal standardization

Signal standardization is by far the most widely used approach among calibration update methods. It was applied to drift removal and calibration transfer from one instrument to the other for both electronic nose and electronic tongue. Signal standardization consists in using a relationship between sensor responses in the initial (at the time of calibration) and new conditions in standardization sample set for correction of the data measured in unknown samples in new conditions. Methods used for data standardization differ in the way the relationship between two sets of sensor signals is calculated (Wang et al., 1991; Bouveresse et al., 1996; Feudale et al., 2002). Single wave standardization (SWS) calculates relationship between each signal individually, piecewise direct standardization (PDS) between groups of signals and direct standardization (DS) between all signals. Both SWS and PDS are linear methods, i.e., they account for the linear shifts of the sensor signals. PDS was proposed as an improvement over SWS with the rational that spectroscopic signals at the adjoin wavelengths are highly correlated, which allows to take into account not only vertical but also horizontal shifts, i.e., shifts of the wavelengths. Both SWS and PDS have been applied to the standardization of the potentiometric sensor data and PDS was found to produce better results (Sales et al., 1999). However, responses of sensors in an array can be independent or partially correlated depending on the array composition, sensor arrangement in the array and analyzed samples, which contradict rational of PDS of high correlation between adjoin signals. Considering this, standardization method that corrects all signals simultaneously, the DS, is more commonly used for the calibration transfer for sensor arrays. Relationship between signals measured in the initial and new conditions can be calculated by means of various multivariate techniques, such as the Multiple Linear Regression (MLR), Partial Least Square 2 regression (PLS2) or auto-associative artificial neural network (ANN), among others.

Calibration transfer from one “master” to four “slave” identical electronic noses equipped with 8 polymer coated QMB sensors has been performed using univariate and multivariate methods, namely linear regression and PLS2 regression (Tomic et al., 2002). Measurements were carried out in the individual solutions of anisole, cyclohexanone, propanol, and toluene during 50 weeks. Calibration transfer was done using the first 72 measurement points acquired over the period of ca. 1 month for each instruments with the rest of the data used for testing. Both linear regression and PLS2 were demonstrated to be successful in removing shifts between instruments according to the RMSEP of the predicted sensor response.

Calibration transfer from one electronic nose to the other using robust regression has been proposed in Deshmukh et al. (2014). Electronic nose instruments were identical and each was equipped with an array of 6 MOX sensors. Calibration model was calculated using back-propagation ANN and measurements in gas samples collected at the pulp and paper production sites. Transfer of the calibration model to the other instrument was done using 27 transfer samples comprising 27 mixtures of four target compounds (hydrogen sulfide, dimethyl sulfide, dimethyl disulphide e methyl mercaptan).

Comparison of the four methods of data standardization has been done using two electronic noses of different models each equipped with identical 12 conducting polymer sensors and measurements in milk samples stored for different periods. Data standardization was done by ANN, MLR, and least squares individually for each sensor with and without intercept, of which MLR produced the best results for the storage time prediction (Balaban et al., 2000).

Calibration transfer between two electronic noses employing different types of sensors, one with QMB sensors and another with conducting polymer sensors, has been described (Shaham et al., 2005). Measurements in vapors of 23 organic compounds were used for both calibration and data standardization. Performance in data standardization of MLR, PLS2, Principal Component regression (PCR), ANN and a method introduced in this work called Tessellation-based linear interpolation (TLT) was compared. The TLT is a local method that uses for prediction only calibration samples near unknown one. The TLT consists of two stages: tessellation and prediction. First, tessellation of the calibration data, i.e., sensor responses, X, for which class labels Y are known, is performed. Tessellation is done in such a way that all vertices of all simplexes are calibration set X points. Prediction of class membership of a new sample is done by first locating simplex enclosing vector of sensor responses and calculating barycentric coordinates of this sample relative to the vertices of simplex that encloses it. The barycentric coordinates of a point p within a simplex can be defined as weights, which, if placed at the simplex's vertices, will have their center of mass coincide with p. After normalization of sum of coordinates to 1, unique coordinates are obtained that are used as interpolation weights. The value of the Y is when predicted as the average of Y values of the simplex's vertices, weighted according to the barycentric coordinates. Among all data standardization methods, ANN was found to be the most effective for the studied data. Unfortunately, no comparison with uncorrected data was shown. Another observation is that mapping from quartz microbalance sensor array to conducting polymer ones was more complicated and yielded higher classification errors than vice versa.

In the works described above successful calibration transfer between electronic noses using data standardization has been demonstrated, though relatively large data sets (Balaban et al., 2000; Tomic et al., 2002; Deshmukh et al., 2014) or even entire calibration sets were necessary for the efficient calibration transfer (Shaham et al., 2005).

Comparison of different approaches to the data standardization for the calibration transfer from one sensor array to the other and for calibration update for the same sensor array were reported in Fernandez et al. (2016) and Fonollosa et al. (2016).

In the first series of experiments, measurements were made with five identical arrays of 8 MOX sensors in the individual vapors of ethanol, ethylene, carbon monoxide, or methane, each at 10 concentration levels (Fonollosa et al., 2016). Calibration transfer from the “master” to four “slave” instruments has been carried out as well as calibration update for the same instrument. Calibration was done using measurements from the same measuring session, of which 20 samples were used for calibration and other 20 as tests. Standardization data set comprised 2 concentration levels for each gas, i.e., 8 samples in total Four methods were evaluated for both calibration transfer and update: DS and PDS, both employing PLS2 regression for mapping, OSC and Generalized Least Squares Weighting (GLSW). GLSW is a data selection method as it identifies and “shrinks” instrument channels (sensors or sensor response features) that are responsible for the main sources of variance between initial and new conditions (Martens et al., 2003).

In the second series of experiments, 12 identical arrays composed of three MOX sensors were used for measurements in individual vapors of ethanol, acetone and butanone at 7 concentration levels. Measurements at 0 °C were considered as initial condition and used for calibration while measurements at ±10°C, ±20°C, ±30°C, ±40°C, and ±50°C were considered new conditions. Data correction is performed for an increasing number of transfer samples, from 2 to 12 (Fernandez et al., 2016). In both experiments data standardization improved performance of the calibration model with DS and PDS producing lower prediction errors for the new conditions/new instrument. It was also found that PDS needs less standardization samples to achieve lower error, i.e., 5 vs. 11 necessary for DS (Fernandez et al., 2016).

A combination of two standardization methods–Windows Piecewise Direct Standardization (WPDS) and Standardization Error based Model Improvement (SEMI) was proposed for the electronic nose calibration transfer in Yan and Zhang (2015). WPDS is a modification of PDS that weighs a subset of initial sensor signals used for standardization of new signals. Data standardization by WPDS was implemented using ridge regression algorithm. SEMI works similarly to GLSW by weighing down variables that had highest standardization errors, i.e., sensors that drifted most, before feeding standardized new data to the calibration model for prediction. This approach was tested on the data from three identical portable electronic noses equipped with 8 MOX sensors, one of which was considered “master” instrument. Seven groups of samples were measured: acetone, hydrogen and ammonia at different concentration levels, air exhaled by healthy people and air exhaled by healthy people and spiked by three aforementioned compounds, 248 samples in total. All available measurements were used for calculating classification and calibration model for prediction of gas concentrations. Six samples, three individual compounds at two concentration levels, were used as transfer set for calibration transfer from “master” to two “slave” instruments. Combination of WPDS and SEMI was effective for the calibration transfer particularly in the case of the regression models, where RMSEP of concentrations decreased, in some cases 3-fold compared to uncorrected data. Improvement of the correct classification rate for classification models was more modest, which indicates higher tolerance of the classification model to drift.

Application of the Robust Weighted Least Square (RWLS) to the data standardization was described in Zhang et al. (2011). RWLS belongs to the robust regression algorithms that owe their name to the property of being less sensitive or more “robust” in the presence of outliers in the data. Detailed description of RWLS algorithm implemented in Zhang et al. (2011) can be found elsewhere (Heiberger and Becker, 1992). Calibration transfer was done from one “master” to five “slave” electronic noses, all equipped with 3 MOX sensors, using measurements in the individual vapors of formaldehyde, benzene and toluene at different concentration levels. Transfer data comprising 5 samples of formaldehyde, which was considered a reference gas, were selected by Kennard-Stone algorithm. Data standardization by RWLS allowed to achieve lower concentration prediction errors compared to uncorrected data. It was also observed that efficiency of data standardization varied between “slave” instruments.

Signal standardization was applied to both calibration transfer and update for the electronic tongue sensor system (Panchuk et al., 2016; Debus et al., 2017; Khaydukova et al., 2017a,b).

Calibration update was applied to the potentiometric electronic tongue comprising 10 sensors with plasticized PVC and chalcogenide glass membranes (Panchuk et al., 2016). Measurements were made in the tap water spiked with different amount of cyanobacteria growth media from both nontoxic and toxic, i.e., microcystin producing, strains over the period of 74 days. Tap water and two solutions of inorganic salts in HEPES buffer at different concentration levels, which were used as standardization samples, were measured at each measuring session. Data were standardized by SWS and DS, employing LS regression and MLR for the data mapping, respectively. Both standardization methods were effective in drift removal in classification model, allowing to maintain correct classification rate throughout the experiment. SWS performed better in microcystin quantification achieving lower errors compared to both DS and uncorrected data though ca. 2-fold increase of RMSEP was observed along the time. Surprisingly, no improvement was found for data standardization by DS as RMSEP of microcystin concentration was the same with uncorrected and DS corrected data.

The same two techniques, SWS and DS with MLR and Tikhonov regularization were used for the calibration transfer between two identical arrays of potentiometric sensors (Khaydukova et al., 2017b). Arrays comprising 17 sensors with plasticized PVC membranes was used for measurements in mixed solutions of 6 lanthanides. Ten solutions selected by Kennard-Stone algorithm were used for standardization. DS with Tikhonov regularization performed better producing lowest RMSEP for all tested models, i.e., prediction of sum of all, light and heavy lanthanides, which were close to the errors obtained using the “master” instrument. SWS and DS showed unstable behavior with DS producing highest errors for prediction of sum of all and light lanthanides and SWS producing highest errors for the prediction of heavy lanthanides and an error slightly lower than DS for the prediction of light lanthanides.

Results reported in Panchuk et al. (2016) and Khaydukova et al. (2017b) indicate that performance of data standardization methods is dependent on the data and, probably, composition of standardization data sets.

An ambitious task of calibration transfer between two different types of sensor systems was described in Khaydukova et al. (2017a). Two electronic tongues, potentiometric one comprising 26 sensors with plasticized PVC and chalcogenide glass membranes and voltammetric one comprising 4 carbon paste electrodes modified with metal nanoparticles, were used for measuring 8 samples of must of different grape varieties. Three samples were selected for the calibration transfer using Kennard-Stone algorithm. Transfer of the PLS regression models for the prediction of tartaric acid content, pH and total phenolics was done by DS. Transfer of the calibration model from voltammetric to potentiometric sensor array worked better than vice versa, which is in agreement with the results reported for the two electronic noses based on different types of sensors (Shaham et al., 2005). RMSEP values close to the “master” electronic tongue were obtained for tartaric acid and total phenolics when calibration was transferred from voltammetric electronic tongue to the potentiometric one. In other cases calibration transfer was not successful. The authors point to the importance of the standardization samples for calibration transfer to work considering that Kennard-Stone algorithm may be not be optimal for this purpose. Another culprit can be limited number of calibration samples that were available in this work.

### Model expansion

This group of methods is based on the expansion of calibration model by joining initial calibration data set and measurements made in new conditions in the subset of standardization or transfer samples, and recalculating calibration model. In this way, new sources of variance are incorporated in the updated calibration model, which allows to decrease prediction errors for the samples measured in new conditions.

Application of three methods of calibration model expansion, namely weighting, Tikhonov regularization and Joint-Y PLS, to the calibration update of potentiometric sensor array has been reported in Rudnitskaya et al. (2017).

Weighting is the most straightforward approach to the model expansion consisting in simply adding newly measured standardization samples to the calibration data set and re-calculating the model (Stork and Kowalski, 1999; Capron et al., 2005).

Initial calibration model can be expressed as:

(4.3) $${\text{y}}_{\text{i}}={\text{X}}_{\text{i}}{\text{b}}_{\text{i}}+\text{e},$$

where X_i_ is a matrix of sensor responses and y_i_ is concentrations.

Regression coefficients *b*_*i*_ are calculated according the following expression:

(4.4) $${\text{b}}_{\text{i}}={\text{X}}_{\text{i}}^{\prime }{\text{y}}_{\text{i}}$$

Model update is performed by adding measurements in standardization samples made in new conditions to the initial sensor response matrix and recalculating calibration model according to the equation:

(4.5) $${\text{b}}_{\text{n}}={\left[\begin{array}{c} {\text{X}}_{\text{i}}\\ {\text{WX}}_{\text{n}} \end{array}\right]}^{\prime }\times \left[\begin{array}{c} {\text{y}}_{\text{i}}\\ {\text{Wy}}_{\text{n}} \end{array}\right],$$

where X_n_ is a matrix of responses in the transfer samples, y_n_ is respective reference values and W is a weighting factor that may be applied to the transfer data set. Number of samples in the transfer data set is typically significantly smaller than in the initial calibration set. Thus, increasing weight of added samples is necessary to avoid that initial calibration data dominate updated model. Sample weighting is usually done by including multiple copies of the standard update samples. Value of W has to be determined empirically.

Tikhonov regularization is a variant of a ridge regression adapted to the calibration update purposes (Kalivas et al., 2009; Kunz et al., 2010).

Standard form of TR or ridge regression can be expressed as follows:

(4.6) $$\left[\begin{array}{c} \text{X}\\ \lambda \text{I} \end{array}\right]\text{b}=\left[\begin{array}{c} \text{y}\\ 0 \end{array}\right],$$

where I is identity matrix and λ is a regularization meta-parameter.

Regression coefficients b can be calculated according to the following equation:

(4.7) $$\hat{\text{b}}={\left({\text{X}}^{\prime }\text{X}+\lambda^{2}\text{I}\right)}^{-1}{\text{X}}^{\prime }\text{y}$$

Modification of Tikhonov regularization to make it applicable to the calibration update consists in introduction of an additional meta parameter τ:

(4.8) $$\left[\begin{array}{c} {\text{X}}_{\text{i}}\\ \tau \text{I}\\ {\lambda \text{X}}_{\text{n}} \end{array}\right]{\text{b}}_{\text{n}}=\left[\begin{array}{c} {\text{y}}_{\text{i}}\\ 0\\ {\lambda \text{y}}_{\text{n}} \end{array}\right]$$

Parameter τ enhances the degree of nonsingularity of the covariance matrix in the inverse operation. Regression coefficients b_n_ for the updated calibration model can be calculated using the following expression:

(4.9) $${\hat{\text{b}}}_{\text{n}}={\left({\text{X}}_{\text{i}}^{\;\prime }{\text{X}}_{\text{i}}+\tau^{2}\text{I}+\lambda^{2}{\text{X}}_{\text{n}}^{\;\prime }{\text{X}}_{\text{n}}\right)}^{-1}\left({\text{X}}_{\text{i}}^{\;\prime }{\text{y}}_{\text{i}}+\lambda^{2}{\text{X}}_{\text{n}}^{\;\prime }{\text{y}}_{\text{n}}\right).$$

Both parameters λ and τ need to be optimized.

Joint-Y Partial Least square regression (JYPLS) has been developed to solve a product transfer problem from one plant to the other while maintaining the same quality of the final product (Jaeckle and Macgregor, 1998; García Muñoz et al., 2005). When applied to calibration update. JYPLS consists in modeling common latent variable space of the response matrices in initial (i) and transfer (n) calibration samples, X_i_ and X_n_, and corresponding concentrations, Y_i_ and Y_n._. JYPLS models joint Y matrix combining Y_i_ and Y_n_ using matrices X_n_ and X_n_ (García Muñoz et al., 2005):

(4.10) $${\text{Y}}_{\text{j}}=\left[\begin{array}{l} {\text{Y}}_{\text{i}}\\ {\text{Y}}_{\text{n}} \end{array}\right]=\left[\begin{array}{l} {\text{T}}_{\text{i}}\\ {\text{T}}_{\text{n}} \end{array}\right]{\text{Q}}_{\text{ j}}^{\prime }+{\text{E}}_{{\text{Y}}_{\text{j}}}$$

(4.11) $${\text{X}}_{\text{i}}={\text{T}}_{\text{i}}{\text{P}}_{i}^{\;\prime }+{\text{E}}_{{\text{X}}_{\text{i}}}\;{\text{X}}_{\text{n}}={\text{T}}_{\text{n}}{\text{P}}_{\text{n}}^{\;\prime }+{\text{E}}_{{\text{X}}_{\text{n}}}$$

(4.12) $${\text{T}}_{\text{i}}={\text{X}}_{\text{i}}{\text{W}}_{\text{i}}^{\text{*}}\;{\text{T}}_{\text{n}}={\text{X}}_{\text{n}}{\text{W}}_{\text{ n}}^{*}$$

(4.13) $${\text{W}}_{\text{i}}^{*}={\text{W}}_{\text{i}}{\left({\text{P}}_{\text{i}}^{\prime }{\text{W}}_{\text{i}}\right)}^{-1}\;{\text{W}}_{\text{n}}^{*}={\text{W}}_{\text{n}}{\left({\text{P}}_{\text{ n}}^{\prime }{\text{W}}_{\text{n}}\right)}^{-1}$$

Where P_i_, W_i_, T_i_, P_n_, W_n_, and T_n_ are weights, loadings and scores for the X_i_ and X_n_ matrices (,) that have the same interpretation as in the PLS regression model, and Q_J_ is a common loading matrix of Y. JYPLS is very flexible as response matrices X_i_ and X_n_ can have different number of both variables and samples and matrices Y_i_ and Y_n_ can have different number of samples. However, matrices Y_i_ and Y_n_ must have the same number of variables and matrices X_i_ and X_n_ should have the same covariance structure.

Comparison of model expansion methods, weighting, Tikhonov regularization and JYPLS, and data standardization methods, slope and bias correction of predicted values and DS with PLS2 regression and ANN for data mapping, has been done for the electronic tongue (Rudnitskaya et al., 2017). An array of 7 potentiometric sensors with chalcogenide glass and plasticized PVC membranes was used for measurements in copper, lead and cadmium mixed model solutions during 3 months. Calibration model was calculated using samples measured during first experimental session and used for prediction of copper and lead concentrations in samples measured in consequent sessions. A set of transfer samples, from 4 to 7, was selected using Kennard-Stone algorithm. Model expansion methods generally performed better achieving lowest RMSEP of lead concentrations and the same RMSEP of copper concentration but with smaller number of transfer samples compared to the data standardization.

Furthermore, JPLS was used to account for the matrix effect for the potentiometric electronic tongue (Cruz et al., 2018). Electronic tongue constituted by 6 sensors with plasticized PVC membranes was calibrated in the mixed solutions of four paralytic shellfish toxins. Afterwards, 4 mixed solutions prepared in bivalve extracts were used as transfer samples for the calibration recalculation. Updated calibration model was applied to the quantification of three toxins in contaminated bivalve extract. Results obtained using updated calibration model were close to the reference method, while without update calibration model was unusable.

Model expansion by a variant of ridge regression called by the authors transfer sample-based coupled task learning (TCTL) has been reported for an electronic nose (Yan and Zhang, 2016a). Two tasks were addressed: calibration transfer using data set described in Yan and Zhang (2015) and calibration update using long-term drift data set described in Vergara et al. (2012). Calibration update for the long-term drift data set was done using 10 transfer samples selected using Kennard-Stone algorithm, as it was found that smaller number of samples did not ensure the best performance. For both tasks and data sets, TCTL allowed to obtain better results compared to the uncorrected data and performed similarly to the combination of variable standardization with SEMI (Yan and Zhang, 2015), TCTL with SEMI and DAELM (Zhang and Zhang, 2015).

An important issue in model expansion methods is validation of the updated calibration models for optimization of the model parameters. Cross-validation is not a viable option as number of transfer samples is typically limited while validation using initial calibration samples would not reflect model performance in new unknown samples. Several approaches to model diagnostics that do not require use of validation data set has been proposed to deal with this issue. These tools mainly focus on finding a trade-off between bias and variance of the updated calibration model, i.e., finding number of latent variables or model parameter values (transfer sample weights, Tikhonov regularization parameters λ and τ) that minimizes both. Graphic diagnostic tools such as plots of b-coefficients errors of the updated calibration model vs. RMSEC or RMSE in calibration samples vs. RMSE in transfer samples, have been demonstrated to be efficient (Stork and Kowalski, 1999; Green and Kalivas, 2002; Kalivas et al., 2009).

### Selection of standardization samples

In practice it is preferable to avoid using large data sets for the data standardization or calibration transfer. Thus, efforts were directed to decrease number of standardization samples. This can be achieved by careful selection of standardization samples with the aim to identify samples describing enough variation to allow successful calibration transfer while keeping number of samples necessary to measure in new conditions or by new instrument to the minimum. In some instances standardization samples can be selected on the basis of the previous knowledge or convenience, i.e., each analyte at two concentration levels, when the task is discrimination of individual gases' vapors at different concentration levels. In other cases, leverage (Hoaglin and Welsch, 1978) and Kennard-Stone algorithm (Kennard and Stone, 1969) were proposed for identification of the of the most relevant samples for the calibration transfer. Leverage matrix is calculated as a covariance matrix of the sensor array mean-centered responses. Maximum diagonal elements of the leverage matrix correspond to the most relevant samples in the training data set. Kennard-Stone algorithm is commonly used for selection of samples uniformly distributed over the object space. This is sequential procedure consisting of selecting as the next sample the one that is most distant from those already selected. Two samples that are the most distant from each other serve as a starting point. The distance is usually the Euclidean distance.

### Adaptive learning

Adaptive drift correction methods are based on the idea of continuous update of the classifier using unknown samples measured during routine functioning of the sensor array. This approach is attractive for practical applications as it does not require reference samples beyond the calibration data set and does not require long-term measurements as drift modeling methods. At the first stage calibration is performed by using a set of calibration samples with known class membership to calculate and optimize classification model. In the following testing stage new unknown samples are used for correction/recalculation of the classification model after they have been assigned to the class. Adaptive drift correction can be performed in supervised, unsupervised and semi-supervised mode depending on the employed method.

First implementations of adaptive drift correction used unsupervised neural network—Self-Organizing Map (SOM) (Di Natale et al., 1995; Marco et al., 1998). SOM consists of a rectangular single layer of neurons, whose weight vectors have the same dimensionality as input data (Kohonen, 1996). During calibration step a known sample is presented to the net and distances between this sample and all neurons are calculated using Euclidean or other metrics. Weights of the winning neuron, i.e., neuron closest to the particular calibration sample, and its neighbors are updated to decrease even further their distance to the calibration sample. Learning rate decreases monotonically with the increase of the neuron distance to the winning neuron and along the training. Trained network forms clusters of neurons corresponding to the same class, i.e., to the similar calibration samples. This process is unsupervised, however, after its completion, user intervention is necessary to label clusters according to the classes and define criterion to avoid cluster overlapping, i.e., allocate neurons corresponding to more than one class. During routine operation, training of the SOM can continue with a slow learning rate to account for the sensor drift and resulting cluster displacement. Continuously adapting SOM has been shown to be more robust in the presence of the drift compared to the static one (Marco et al., 1998).

Use of multiple self-organizing maps or mSOM, one for each modeled class, has been proposed with the aim to increase user influence over training process (Distante et al., 2002; Zuppa et al., 2004). mSOM have been tested using data from an array of 32 polymeric conducting sensors measured in 6 gases (acetonitrile, methanol, propanol, acetone, butanol, and water) over the period of 4 weeks. Use of continuous net training permitted to decrease error rate from 9% to less than 3%.

Similar modification of unsupervised technique for the purpose of semi-supervised classification has been proposed for the other type of the network—Artificial Immune Network (AINET). AINET is an algorithm inspired by the adaptive immune system (de Castro and von Zuben, 2002). Modified algorithm called Adaptive Artificial Immune Network (A^2^INET) consists in training separate AINET for each class (Martinelli et al., 2013, 2014). Calibration starts by initiation of a set of processing units or network cells. Distances or affinities between network cells and calibration sample are calculated and a cell closest to this sample is selected. Then selected cell is replicated or cloned and cells with less affinity are changed or mutated. Both number of clones to add and mutation rate being are functions of the affinity of particular cell to the calibration sample. Cells with less affinity are eliminated and a pool of the cells is updated. Detailed description of both original AINET and adapted algorithms can be found in de Castro and von Zuben (2002) and Martinelli et al. (2013). For the purpose of pattern recognition outputs of the trained network are used as inputs into classifier such as e.g., k nearest neighbors (k-NN). A^2^INET is continuously adapted during sensor operation as after new unknown sample is assigned to the class, it is used to clone and mutate network cells.

A^2^INET performance in drift compensation has been assessed using synthetic and experimental data. The latter included measurements with an array of four MOX sensors in five individual gasses (acetaldehyde, acetone, ammonia, ethanol, and ethylene) during 18 months and in 3 gases (acetaldehyde, ethylene, and toluene) during 12 months. A^2^INET permitted to improve classification rate compared to the standard classifier from 81 to 95% (de Castro and von Zuben, 2002; Martinelli et al., 2013) and from 90 to 99% (Martinelli et al., 2014), respectively. Adaptive classifier was also robust in the presence of artificially added noise and faults.

Algorithm of unsupervised on-line selection of training features (UOL) was described in (Magna et al., 2018). This method performs selection of the features of the sensor response matrix that afford better class separation during initial calibration step and after each new sample is measured. Features here refer to the parameters of the response of QMB sensors, for which frequency shifts at different time periods, response integral at different time frames, etc. are measured. After new sample is measured, feature selection from the calibration set is performed in such a way as to avoid that new sample is considered an outlier (far from all classes) or ambiguous (between several classes). Thus, UOL “adapts” calibration data set to new unknown samples. Selected features are used to recalculate classification model. Any classifier, e.g., LDA, PLS-DA, or k-NN can be used in combination with UOL. Feature selection is done using two criteria, MR and PR. MR is the ratio between Mahalanobis distances from the new sample to the two nearest class distributions M_1_ and M_2_. MR is calculated for each feature *i* according to:

$${\text{MR}}_{\text{i}}={\text{M}}_{\text{1}}\frac{{\text{M}}_{1}}{{\text{M}}_{2}}$$

Feature is not included in the calibration model when MR_i_ is bigger than a fixed value, i.e., 0.9. PR evaluates probabilities of a new sample to belong to known class distribution:

$${\text{PR}}_{\text{i}}={\text{max}}_{\text{j}}\left(\frac{1}{\sigma_{j}\sqrt{2\pi }}\exp\left(-\frac{{\left(\text{x}-\mu_{\text{j}}\right)}^{2}}{2\sigma_{j}^{2}}\right)\right),$$

where μ_j_ and σ_j_ are the standard deviation and the mean of the the ith feature for the jth class. A large value of PR_i_ means that new sample has a low probability to be an outlier for at least one class. Thus, features with PR_i_ lower than certain threshold are rejected.

UOL has been applied to the synthetic and experimental data sets, the latter consisted of measurements made in ethanol, toluene and their mixture with an array of seven QMB sensors with metalloporphyrin coatings. Measurements were carried out during two measuring sessions 45 days apart. For the best performing classification method, LDA, use of unsupervised on-line selection allowed to improve classification rate from 88 to 100%. Classification improvement was even more drastic when noise was added to the data: from 66 to 92% without calibration update to 88–100% after using unsupervised on-line selection.

Adaptive drift correction for back-propagation neural network (BPNN) classifier was implemented using two semi-supervised algorithms: semi-boost and Semi-Supervised Regression with Co-Training (COREG) (De Vito et al., 2012). The crucial step in both algorithms is selection of unlabeled samples from the pool for classifier recalculation. Semi-boost selects unlabeled samples with highest relevance, which is estimated by taking into account their classification confidence and the presence of labeled samples in their neighborhood. Thus, COREG algorithm selects unlabeled samples that decrease classification error for the calibration data set when included in it. Both methods have shown improvement of the correct classification rates compared to BPNN without recalculation. Semi-boost classifier update applied to the measurements with an array of 5 MOX sensors in head-space of ground coffee samples improved classification rate from 89 to 93%. COREG was used for model correction for 1 yearlong city air pollution monitoring data measured using the same electronic nose system. Performance gain of 11% was obtained when employing optimal data split into 6% of the data as calibration, 10% as unlabeled sample pool and the rest as test samples.

A method based on system identification theory that models responses of each of the individual sensors of the array in new samples using responses of the other sensors in new and previously measured samples has been proposed (Holmberg et al., 1996, 1997). Dynamic sensor response is described by the linear Box-Jenkins model in the following form:

$${\hat{\text{y}}}_{\text{i}}\left(\text{t}\right)=\text{G}\left(\text{q},\theta^{\text{A}}\right)\text{u}\left(\text{t}\right)+\text{v}\left(\text{t}\right)$$

where $\hat{y_{i}}\left(t\right)\;$is the value of the modeled output of the sensor *i* in discrete time; u(t) are the inputs (signals from the other sensors of the array), G is the linear function of the sensor dynamics; q is the time shift operator, qy(t) = y(t+ 1); Θ^A^ are parameters of the model of the class A; and v(t) is disturbance or noise. Model parameters Θ for each class are estimated using calibration data set. When new (unknown) sample is measured, estimates of each sensor response in this sample ŷ(*t*) are calculated using actual and previous responses of all other sensors of the array for all possible classes (gases). Overall square error E is calculated according to an expression:

$$\text{E}=\sum_{i=1}^{\text{N}}{\left({\text{y}}_{\text{i}}\left(\text{t}\right)-{\hat{\text{y}}}_{\text{i}}\left(\text{t}|{\hat{\theta }}_{\text{i}}^{\text{A}}\right)\right)}^{2}$$

Unknown sample at a time t is assigned to the class, for which the lowest error E was found.

At the same time with each new measurement, classification models are updated using recursive least squares (RLS) algorithm, which assigns exponentially decreasing weights to the older measurements:

$${\hat{\theta }}_{\text{i}}^{\text{A}}\left(\text{t}\right)={\hat{\theta }}_{\text{i}}^{\text{A}}\left(\text{t}-1\right)+{\text{L}}_{\text{i}}\left(\text{t}\right)\varepsilon_{\text{i}}\left(\text{t}\right),$$

where ε_i_(t) is the prediction error for the sensor i calculated according to the previous equation and L_i_(t) is a gain vector estimated by the RLS algorithm. To avoid updating wrong model due to misclassification of the new (unknown) samples, model parameters are updated only in the case of significant difference between the prediction error of the model of the recognized gas and the prediction errors of the other models. This approach has been applied to the data set consisting of the measurements of three MOX sensors in 1-propanoI, 2-propanol, 1-butanol and 2-butanol during 45 days. Measurements collected during the first 10 days (150 measuring cycles) have been used for calculating classification models, while measurements collected during consequent 35 days (730 measuring cycles) were used for model testing and update. Adaptive model displayed lower prediction errors compared to the static model with average classification rates 91 and 85%, respectively (Holmberg et al., 1997).

An approach named classification ensembles was proposed for continuous update of the calibration model during functioning of the electronic nose system (Vergara et al., 2012). First, a Support Vector Machine (SVM) classifier is trained on a set of calibration data. When the next batch of calibration data is available, the next classifier is trained and the final classifier ht+1(x) at time step (t + 1) is a weighted combination of all classifiers. Thus, drift correction is performed by gradually including it in the calibration model. Classifier ensembles were applied to the very large data set consisting of measurements in 6 individual gases (ammonia, acetaldehyde, acetone, ethylene, ethanol, and toluene) at different concentration levels by an array of 16 MOX sensors during 36 months. Classification ensembles were shown to be effective as a drift reduction strategy though the more time elapsed between the last calibration and new unknown samples, the more classification rate deteriorated.

Domain regularized component analysis (DRCA) has been proposed for the adaptive drift correction in Zhang et al. (2017). DRCA finds a common subspace for both reference and new data. Its algorithm can be summarized as follows:

1. Calculate matrix $\text{A}={\left(\left(\mu_{\text{r}}-\mu_{\text{n}}\right){\left(\mu_{\text{r}}-\mu_{\text{n}}\right)}^{\prime }\right)}^{-1}\left({\text{X}}_{\text{r}}{\text{X}}_{\text{r}}^{\;\prime }+{\lambda \text{X}}_{\text{n}}{\text{X}}_{\text{n}}^{\;\prime }\right),$, where μ is a mean vector, X–matrix of sensor responses, indexes r and n refer to reference and new data, correspondingly. λ is a regularization parameter, which is used since less new data are typically available compared to the reference data.
2. Perform eigenvalue decomposition of the matrix A and consider eigenvector corresponding to the first d largest eigenvalues an optimum subspace: P = [p_1_, p_2_, … p_d_].
3. Correct data by calculating subspace projection: ${\text{X}}_{\text{r}}^{\;\prime }={\text{P}}^{\prime }{\text{X}}_{\text{r}}$ and ${\text{X}}_{\text{n}}^{\;\prime }={\text{P}}^{\prime }{\text{X}}_{\text{n}}$.

Performance of DRCA combined multi-class SVM with RBF kernel was evaluated using publicly available data set (Ziyatdinov et al., 2010) and it compared favorably with other classification and drift correction approaches.

Calibration model expansion by inclusion of the transfer samples in the calibration model was described in Zhang and Zhang (2015). Two approaches based on extreme learning machines or back-propagation neural network were employed. The first one named source domain adaption extreme learning machine (DAELM-S) uses transfer samples for regularization or update of the calibration model. The second one named target DAELM or DAELM-T works similarly to a semi-supervised adaptive neural network described in (De Vito et al., 2012; Martinelli et al., 2014). Both algorithms were shown to be more successful in drift reduction compared to CC by PCA and Support Vector Machine classification models as they maintained correct classification rate close or above 90%. It is worth to note that relatively large number of transfer samples were necessary for these algorithm functioning: 20 to 30 for DAELM-S and 40 to 50 for DAELM-T.

An ANN with three hidden layers, which combines drift removal and calibration model update using both new unknown and transfer samples has been described in Yan and Zhang (2016b). This method named drift correction autoencoder (DCAE) includes the following steps:

1. The first denoising layer is pre-trained with new unknown data (or data measured on the “slave” instrument) in unsupervised mode followed by fine-tuning of the network weights using calibration data set (or data measured on the “master” instrument.
2. The second layer is initialized using weights of the denoising layer.
3. The domain vector d (d ∈ X) is created for each calibration, transfer and unknown sample such as d_i_ = 1 if the sample is from the ith device and 0 otherwise. The acquisition time t can also be added into d.
4. The second layer is trained to minimize the expression $\sum_{\text{p}=1}^{\text{P}}{\left\|\text{f}\left({\text{x}}_{\text{p}}^{\text{c}}{\text{d}}_{\text{p}}^{\text{c}}\right)-\text{f}\left({\text{x}}_{\text{p}}^{\text{n}}{\text{d}}_{\text{p}}^{\text{n}}\right)\right\|}^{2}$, where P is the number of transfer samples, x^c^ and d^c^ are transfer samples from the calibration data set or “master” instrument and its respective domain vector, and x^n^ and d^n^ are transfer samples measured at new conditions or “slave” instrument and their respective domain vectors.
5. Using output of the second layer, the third layer is trained as classifier using calibration data set and consequently used to predict class membership for the new unknown data.

DCAE performance was evaluated using public data set described in Vergara et al. (2012) DCAE performed similarly to the other drift reduction methods such as CC by PCA, OSC and classification ensembles for the second data batch, i.e., data measured in the month following calibration. However, contrary to the other methods DCAE was able to maintain this performance for the consequent batches due to the use of transfer samples. DCAE also performed slightly better than DAELM-S.

Obvious attraction of adaptive methods of drift reduction and model update is that no reference samples are required, instead unknown samples measured during routine sensor array operation are used. However, no strategy has been proposed to deal with the situation when due to the sensor drift or condition change newly measured samples began to be allocated to the wrong classes. It also should be noted that the best performance of the adaptive correction is achieved when all sensors of the array display similar behavior with respect to drift.

## Global models

Instead of being modeled, known sources of variation can be also included in the calibration model, which in this case becomes global or general calibration. Combination of the data pre-treatment and variable selection by fuzzy inference system with linear multivariate regression was proposed to account for the effects of humidity on the response of an array of gas MOS sensors (Šundić et al., 2002). Measurements with an array of 5 sensors were carried out in carbon monoxide, methane and their mixtures at three humidity levels. Nonlinearity of the sensor responses caused by varying humidity as well as sensor cross-sensitivity at low gas concentrations could be taken into account by the fuzzy inference algorithm.

Global calibration can be applied to the calibration transfer between electronic nose instruments, in which case source of undesirable variation is differences in response characteristics between sensors of the same composition (Solórzano et al., 2018). General calibration model is calculated using measurements made with several replicas of sensor array and is expected to include variations between different sensor arrays of the same composition. This approach was evaluated on 5 arrays constituted by 24 MOX sensors, which were used for measurements in six gases (acetaldehyde, methane, ethanol, propane, nitrogen dioxide, and carbon monoxide) at 3 concentration levels each at varying humidity. General classification models were calculated using multiclass Partial Least Squares–Discriminant Analysis (PLS-DA), followed by k-NN in the latent variable (LV) subspace. Calibration and validation data sets were composed by the measurements of 4 sensor arrays, while measurements with fifth were used to test classification model performance. While individual calibration requires less samples and gives better prediction results compared to the general calibration, the latter is capable to provide significant cost-reduction for mass-produced sensor array ensuring acceptable performance.

## Conclusions

Calibration update is essential for the practical use of the electronic nose and electronic tongue sensor systems. Several methods discussed in this review have been successfully applied to tackle issues of temporarily sensor drift, matrix effects or calibration transfer between instruments. Performance and consequently choice of the calibration update method depends on the data at hand, i.e., on the behavior of the particular sensors in analyzed samples. In practice multivariate calibration update methods may be preferable to the drift modeling as they require only few transfer samples to be measured regularly to maintain calibration model indefinitely. Special attention should pain to the provision of the adequate transfer samples matching matrix of the analyzed media and with reproducible compositions.

As transfer samples in some cases can be of limited availability or have high costs, even if only few of them are necessary, adaptive drift correction methods may be of interest. It is difficult to envisage that adaptive correction can function without any standard samples at all after calibration completion, but it may serve as a mean to decrease even further number of transfer samples or frequency, at which they need to be measured.

Though drift reduction and calibration update are very important issues for practical applications of the sensor systems, they are not routinely used yet. With exception of Component Correction and Direct Standardization, a typical situation for the most methods described in the review is that they were reported only once in an article dedicated to a novel approach to the calibration update, which was tested on the available data set, often public or artificial one. It is important to note that nothing precludes application of methods developed for one type of sensor system to the other.

Finally, while a wide number of algorithms of both drift reduction and calibration update were described, they were mostly tested in the model samples. Only few works dealt with analysis of the real world samples and none of the methods was tested in the real world setting and for long periods of time. More applications of calibration update techniques to the sensor systems deployed in real world scenarios are called for.

## Author contributions

The author confirms being the sole contributor of this work and approved it for publication.

### Conflict of interest statement

The author declares that the research was conducted in the absence of any commercial or financial relationships that could be construed as a potential conflict of interest. The handling editor declared a past co-authorship with the author.
